# The Design and Analysis of Split Row-Column Addressing Array for 2-D Transducer

**DOI:** 10.3390/s16101592

**Published:** 2016-09-27

**Authors:** Xu Li, Yanping Jia, Mingyue Ding, Ming Yuchi

**Affiliations:** Department of Bio-medical Engineering, School of Life Science and Technology, “Image Processing and Intelligence Control” Key Laboratory of Education Ministry of China, Huazhong University of Science and Technology, Wuhan 430074, China; leemax@126.com (X.L.); jiayanping163@126.com (Y.J.); myding@hust.edu.cn (M.D.)

**Keywords:** 3-D ultrasound imaging, row-column addressing, split schemes, imaging simulation

## Abstract

For 3-D ultrasound imaging, the row-column addressing (RCA) with 2*N* connections for an *N* × *N* 2-D array makes the fabrication and interconnection simpler than the fully addressing with *N*^2^ connections. However, RCA degrades the image quality because of defocusing in signal channel direction in the transmit event. To solve this problem, a split row-column addressing scheme (SRCA) is proposed in this paper. Rather than connecting all the elements in the signal channel direction together, this scheme divides the elements in the signal channel direction into several disconnected blocks, thus enables focusing beam access in both signal channel and switch channel directions. Selecting an appropriate split scheme is the key for SRCA to maintaining a reasonable tradeoff between the image quality and the number of connections. Various split schemes for a 32 × 32 array are fully investigated with point spread function (PSF) analysis and imaging simulation. The result shows the split scheme with five blocks (4, 6, 12, 6, and 4 elements of each block) can provide similar image quality to fully addressing. The splitting schemes for different array sizes from 16 × 16 to 96 × 96 are also discussed.

## 1. Introduction

Ultrasound imaging has been widely used to scan the soft tissue of the human body in clinical diagnostics and interventional procedures due to the advantages of no radiation, low cost and bedside availability. However, conventional 2-D ultrasound may suffer from the subjectivity of diagnostician, which results from the dependence on the experience and knowledge of the diagnostician to manipulate the ultrasound transducer in different angles to acquire a series of images of the pathological area and mentally transform 2-D images into 3-D tissue structures [1]. Moreover, the anatomic information of the tissue is difficult to be re-traced with the movement of the transducer.

3-D/4-D ultrasound imaging can overcome these disadvantages. The 3-D feature provides full volumetric image of an internal organ or tissue either in color or grayscale. 4-D imaging also has the 3-D image feature, with the added benefit of allowing the physician to view the image in real-time. In addition, 3-D/4-D ultrasound can supply accurate spatial location information for clinicians in the application of ultrasound surgery guidance.

3-D ultrasound images can be acquired through two different modes: mechanical scan mode and 2-D array mode. In the former one, the 3-D ultrasound image is reconstructed based on a series of 2-D images, which are captured by a linear array transducer scanning along a predefined route. Normally, the movement is driven by a motor and the corresponding gearing. The frame rate is limited by the relatively slow mechanical movement and long reconstruction process [1].

Comparing with the mechanical scan, 2-D array mode shows advanced features. By transmitting and receiving volumetric information directly, 2-D array makes it possible to acquire real-time volume images without moving the transducer. The first 3-D ultrasound system using a 2-D array transducer was developed by Smith and Von Ramm at Duke University in 1991 [2]. After that, various researches on 3-D ultrasound imaging with 2-D array have been reported [3,4,5,6,7,8]. Nowadays, the 3-D/4-D features with 2-D array are available on most of the high-end ultrasound machines.

For 2-D array mode, the fully sampling 2-D array (FSA) imaging method utilizes all the elements in transmit and receive events. It provides the best image quality for a given array size because the beam can be optimally steered and focused in any direction [7]. However, the large number of elements requires highly advanced fabricating and interconnecting techniques. There are also challenges in acquiring and processing data from a large number of ultrasound channels. Another challenge associated with 4-D ultrasound is the requirement on the frame rate. If the frame rate is 20 volume/s, each volume image must be acquired in 50 ms. To produce a single 90°, 15 cm deep pyramidal volume image, no more than 250 transmit events can be used, which may result in a loss of lateral information.

2-D array design methods seek a proper element distribution and interconnection to maximally lower down the system complexity at the least expense of image quality. Various array design and processing techniques have been proposed for 3-D/4-D imaging, such as minimally redundant array [7], sparse array [8,9,10,11,12], spiral array, synthetic aperture [12,13], phased sub-arrays [14,15], and row-column addressing array [16,17,18,19,20,21,22,23,24,25,26,27].

Sparse array was firstly proposed by Von Ramm et al. to simplify the front-end complexity of 3-D system [9]. It reduces the active channel count based on aperture undersampling. There are two types of sparse array: random sparse array and periodic sparse array. The former breaks the periodic nature of the element distribution of the latter to reduce the grating lobe levels by means of randomly selecting the active elements with a predefined probability. In order to find out the optimized element distribution, a series of optimization methods have been developed such as genetic algorithm [28,29], simulated annealing [30] and linear programming [31].

The spiral array was first proposed by Schwartz et al. [13]. Different from the random array with asymmetric elements, the spiral array arranges elements on an exponential spiral. The spiral array has the advantage of aperiodic nature, which avoids the formation of grating lobes. However, it is difficult to optimize the design with the proposed function. To address this problem, A. Tweedie et al. presented the rotationally symmetric layout [14], which had flatter side lobes than traditional periodic array, while requiring fewer array controller channels.

The phased subarray (PSA) [15,16] was implemented by combining the phased array and the synthetic aperture technique. In this method, a 1-D N-element array is subdivided into *K* overlapping subarrays with *M* elements, which are used to collect a group of beam signals in one firing event. The subarray beam origins are located at the center of the full array. After all, the subarrays are fired, a post processing technique is introduced to form a high-resolution image. For the 2-D array, the combinations of transmit and receive elements may contribute to the same location of effective aperture, resulting in high redundancy. To solve this problem, Karaman et al. presented the concept of minimally redundant array based on effective aperture to obtain real-time volume imaging with acceptable spatial resolution [7].

**Figure 1:**
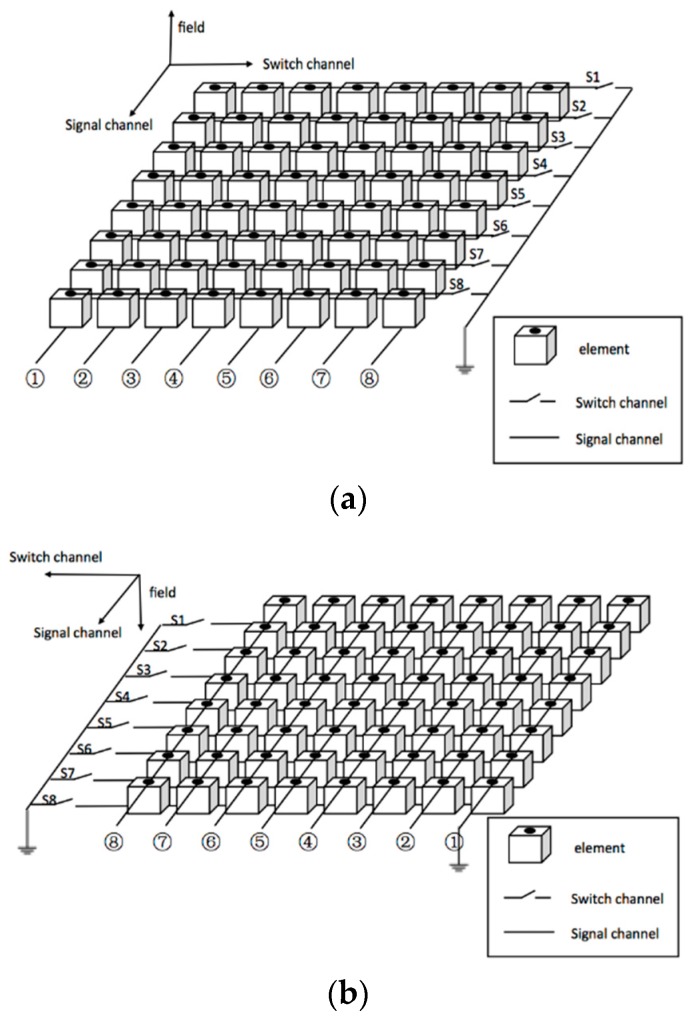
The row-column addressing scheme for an 8 × 8 array: (**a**) obverse side and (**b**) reverse side.

Another effective method to reduce the front-end complexity of 3-D ultrasound system with 2-D array is row-column addressing (RCA) [16,17,18,19,20,21,22,23,24,25,26,27]. The design utilizes a two-layer electrode pattern where the top layer electrodes in the same row or column are connected together with a switch to the ground (switch channel), and the bottom layer electrodes in the same column or row are connected together serving as one signal channel of the system (Figure 1). For an *N* × *N* array, RCA has only *N* signal channels and *N* switch channels rather than *N*^2^ channels for FSA.

**Figure 2:**
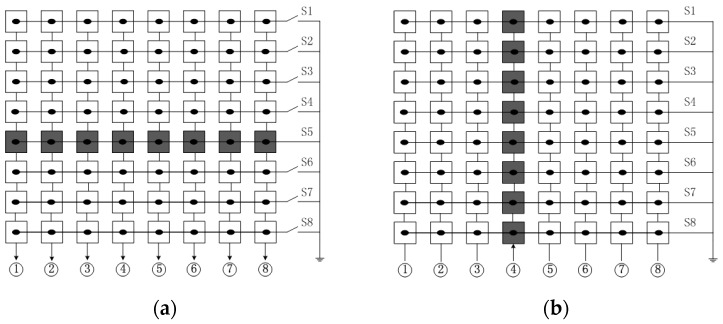
The transmit (**a**) and receive (**b**) activated elements indicated in the grey shading for an 8 × 8 array.

RCA is adopted to simplify the front-end complexity at the cost of signal strength loss, resolution decrease in signal channel direction and secondary lobe amplitude increase. The signal strength loss can be partially compensated for by increasing the transmit power. The reason of the resolution decrease in signal channel direction is because the elements in signal channel direction can only be excited simultaneously to generate planar wave so that the ultrasound beam cannot be focused in the signal direction. Figure 2 shows an example of transmit and receive events with RCA. In Figure 2a, the elements of channel 5 marked with the grey shading are excited simultaneously to generate a planar transmit beam in signal channel direction. In Figure 2b, a focus receive beam can be obtained through beam post-progressing. The two-way radiation pattern for a 2-D array with RCA shows the poor main lobe and side lobe character in signal channel direction. To get similar image resolution as the FSA, the number of elements must be doubled [20], which requires a transducer with bigger size.

In this paper, we propose an improved array inter-connection scheme named split row-column addressing (SRCA) [32]. The connected elements in signal channel direction are divided into several independent blocks to achieve focusing ultrasound beam. The detailed description of the SRCA is given in Section 2. The simulation results in Section 3 show that the image qualities are influenced by the number of independent blocks and the number of element in each block. Certain SRCA design can obtain a radiation pattern similar to FSA. A point target simulation results from a 4 MHz 32 × 32 array with SRCA is also given. Further discussions with various array sizes are presented in Section 4, and Section 5 concludes the paper.

## 2. Method

### 2.1. Splitting Row-Column Addressing (SRCA)

**Figure 3:**
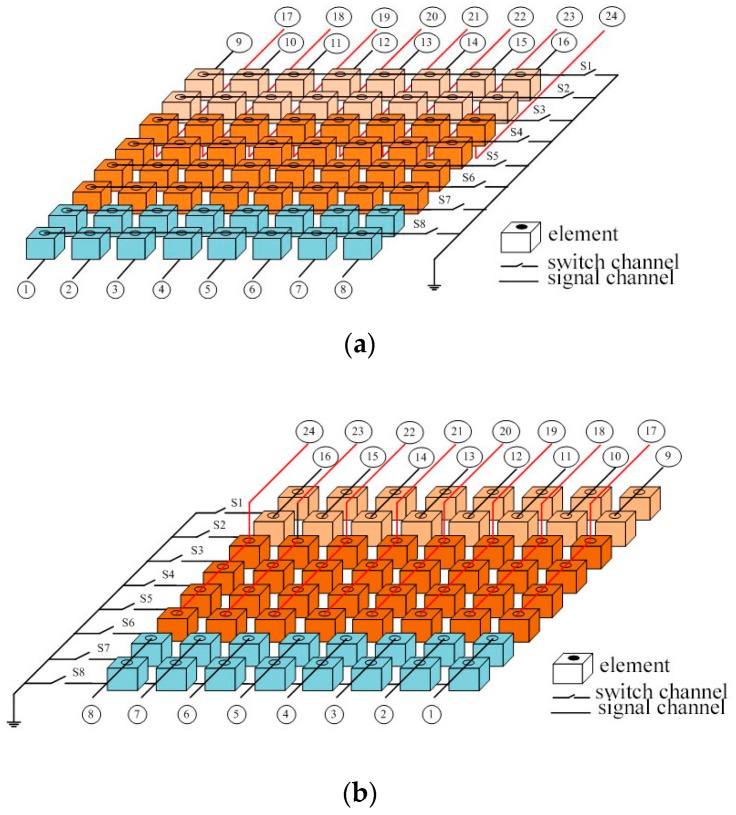
The split row-column addressing scheme for an 8 × 8 array, where the elements in signal channel direction are divided into three blocks: (**a**) obverse side and (**b**) reverse side.

The main idea of SRCA is to divide the elements in signal channel direction into two or more independent blocks. The elements in each block are connected together to one individual signal channel in signal channel direction. In switch channel direction, the element connection scheme is the same as that of the RCA. Figure 3 shows an example of SRCA for an 8 × 8 2-D array. In the signal channel direction, the elements are divided into three blocks which are marked with different colors, and the numbers of elements in the blocks are 2, 4, and 2.

SRCA is designed to improve the image resolution in signal channel direction. This is realized by producing the focusing beam with corresponding delay time for each block in the signal channel direction. The elements in a certain block in signal channel direction are fired simultaneously. For an *N* × *N* array with *K* blocks in signal channel direction, the number of signal channels is *KN*, the number of physical connections is (*K* + 1)*N*, while the corresponding numbers of RCA are *N* and 2*N*, FSA *N*^2^ and *N*^2^.

### 2.2. Two-Way Radiation Pattern of SRCA

**Figure 4:**
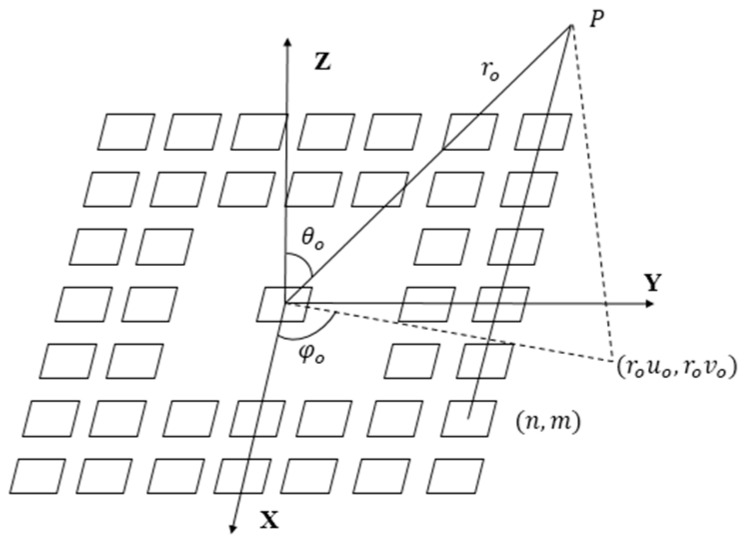
The coordinate system for the *N* × *N* array.

Set the center of the *N* × *N* array to be the origin of the coordinate system (Figure 4).

Assume the polar coordinate of focus point is $P\;(r_{0},\theta_{0},\varphi_{0})$, the projected coordinate on x-y plane is $\left(r_{0}u_{0},r_{0}v_{0}\right)$, where $u_{0}=\sin\theta_{0}\cos\varphi_{0},v_{0}=\sin\theta_{0}\sin\varphi_{0}$. In case of far-field continuous approximation, the two-way radiation pattern of an array with the appointed target located at $\vec{r}=(r,\theta ,\varphi )$ is equal to the product of the Fourier transform of transmit and receive impulse response [33],

(1) $$D_{TR}(\theta ,\varphi )=D_{T}(\theta ,\varphi )D_{R}(\theta ,\varphi )$$

where $D_{T}$ and $D_{R}$ are the impulse responses of the transmit and receive array, respectively. As described in [34], the transmit or receive impulse response $D(\vec{r},t)$ is decided by the travel time $t_{nm}$, which represents the time of sound propagation from the (n,m) the element to the focus point, and the delay time $\Delta t_{nm}$ which represents the delay time of each element to the focus point:

(2) $$D(\vec{r},t)=\sum_{n=1}^{N}\sum_{m=1}^{N}\frac{e^{jw(t-t_{nm}-\Delta t_{nm})}}{r_{nm}}$$

and

(3) $$\begin{array}{l} t_{nm}=\;\frac{\sqrt{{\left(ru-nd\right)}^{2}+{\left(rv-md\right)}^{2}+(\cos\theta r{)}^{2}}}{c}\\ \Delta t_{nm}=\frac{r_{0}}{c}-\frac{\sqrt{{\left(r_{0}u_{0}-nd\right)}^{2}+{\left(r_{0}v_{0}-md\right)}^{2}+(\cos\theta_{0}r_{0}{)}^{2}}}{c}\\ r_{nm}=t_{nm}c \end{array}$$

where c is the speed of sound, $r_{0}$ the distance from the focus point to array center, *d* the element pitch.

Based on Equations (2) and (3), the two-way radiation pattern of *N* × *N* FSA can be deduced by

(4) $$D_{FSA}(\vec{r},t)=(\frac{\sin(\alpha N)}{\sin\alpha }\times \frac{\sin(\beta N)}{\sin\beta })\times (\frac{\sin(\alpha N)}{\sin\alpha }\times \frac{\sin(\beta N)}{\sin\beta })$$

where α=w×du/2c, β=w×dv/2c, and w is the center frequency of the array.

Similarly, the two-way radiation pattern of *N* × *N* RCA is

(5) $$D_{RCA}(\vec{r},t)=\frac{\sin(\beta N)}{\sin\beta }\times (\frac{\sin(\alpha N)}{\sin\alpha }\times \frac{\sin(\beta N)}{\sin\beta })$$

SRCA separates the elements into *K* independent blocks in signal channel direction. Assume the number of elements of each block is $Z_{1},Z_{2},\cdots ,Z_{K}$, the distance from the center of each separated block to the center of the whole array in signal channel direction is $d_{1},d_{2},\cdots ,d_{K}$. The delay time in transmit event from each block to focus point $\left(r_{0},\theta_{0},\varphi_{0}\right)$ can be described as:

(6) $$\Delta t_{Tnm}=\frac{r_{0}}{c}-\frac{\sqrt{{\left(r_{0}u_{0}-nd\right)}^{2}+{\left(r_{0}v_{0}-md\right)}^{2}+(\cos\theta_{0}r_{0}{)}^{2}}}{c}$$

where n=1,2,…,K,m=1,2,…N.

Receive focusing of SRCA is achieved by varying element delays with synthetic technology in row direction, which is same as FSA with the synthetic receive delay

(7) $$\Delta t_{Rnm}=\frac{r_{0}}{c}-\frac{\sqrt{{\left(r_{0}u_{0}-nd\right)}^{2}+{\left(r_{0}v_{0}-md\right)}^{2}+(\cos\theta_{0}r_{0}{)}^{2}}}{c}$$

where n=1,2,…,N,m=1,2,…N.

With Equations (6) and (7), the corresponding two-way radiation pattern for an *N × N* SRCA array with *K* independent blocks in signal channel can be achieved by

(8) $$D_{SRCA}(\vec{r},t)=\sum_{n=1}^{K}e^{jwd_{n}u}\times \frac{\sin(\beta N)}{\sin\beta }\times (\frac{\sin(\alpha N)}{\sin\alpha }\times \frac{\sin(\beta N)}{\sin\beta })$$

Equation (8) indicates that the two-way radiation pattern of SRCA is dependent on the value of *K* and $d_{n}$. Through adjusting the value of *K*, SRCA maintains a balance between the RCA and FSA in image quality and front-end complexity. When *K* equals one, SRCA is the same as RCA, while when *K* is *N*, SRCA turns to FSA. For simplifying the design procedure, in this paper, we mainly study the SRCA with symmetrical scheme. This means, when K≥2 and *K* is an odd number,

(9) $$\begin{array}{l} Z_{1}=Z_{K}\in \left\{1,\cdots ,\left\lceil \frac{N}{2}\right\rceil -\left\lfloor \frac{K}{2}\right\rfloor \right\}\\ Z_{2}=Z_{K-1}\in \left\{1,\cdots ,\left\lceil \frac{N}{2}\right\rceil -(\left\lfloor \frac{K}{2}\right\rfloor -1)-Z_{1}\right\}\\ \cdots \\ Z_{\left\lceil \frac{K}{2}\right\rceil }\in \left\{1,\cdots ,N-2(Z_{1}+Z_{2}+\cdots +Z_{\left\lfloor \frac{K}{2}\right\rfloor })\right\} \end{array}$$

where *N* is the number of elements for an array in signal channel direction. ⌊·⌋ and ⌈·⌉ represent round down and up, respectively. When K≥2 and *K* is an even number,

(10) $$\begin{array}{l} Z_{1}=Z_{K}\in \left\{1,\cdots ,\left\lceil \frac{N}{2}\right\rceil -(\frac{K}{2}-1)\right\}\\ Z_{2}=Z_{K-1}\in \left\{1,\cdots ,\left\lceil \frac{N}{2}\right\rceil -(\frac{K}{2}-2)-Z_{1}\right\}\\ \cdots \\ Z_{\frac{K}{2}}=Z_{\frac{K}{2}+1}\in \left\{1,\cdots ,\left\lceil \frac{N}{2}\right\rceil -(Z_{1}+Z_{2}+\cdots +Z_{\frac{K}{2}-1})\right\} \end{array}$$

## 3. Results

To investigate the influence of *K* and $Z_{i}$ on the image quality, a 32 × 32 array with the center frequency of 4 MHz and the pitch size of 0.5λ = 0.19 mm was adopted for simulation. The two-way radiation pattern with the focus point set at (x,y,z)=(0,0,40) mm was calculated. −6 dB and −20 dB main-lobe widths were measured to indicate the resolution. In particular, −40 dB beam width was added in our paper to show the clutter level. Other parameters related to the image quality such as average side lobe levels (ASLL), peak side lobe level (PSLL) and main lobe-to-side lobe energy ratio (MSR) were also provided. The main lobe is defined in the scope of $\sin^{2}\theta \leq 0.04$. MSR is measured with Equation (11),

(11) $$\mathrm{MSR}=\frac{\sum_{\theta =0{}^{\circ}}^{90{}^{\circ}}\sum_{\varphi =-90{}^{\circ}}^{90{}^{\circ}}{\left|PSF_{m}(\theta ,\varphi )\right|}^{2}}{\sum_{\theta =0{}^{\circ}}^{90{}^{\circ}}\sum_{\varphi =-90{}^{\circ}}^{90{}^{\circ}}{\left|PSF_{s}(\theta ,\varphi )\right|}^{2}}$$

where $PSF_{m}(\theta ,\varphi )$, $PSF_{s}(\theta ,\varphi )$ represent the point spread function of the main lobe beam and side lobe beam, respectively.

Firstly, the influence of $Z_{i}$ was investigated with a fixed *K* = 3. In this paper, only the symmetrical schemes were considered because they could provide symmetrical resolution and contrast along the signal channel direction. Table 1 gives out all the 15 symmetrical split schemes.

**Figure 5:**
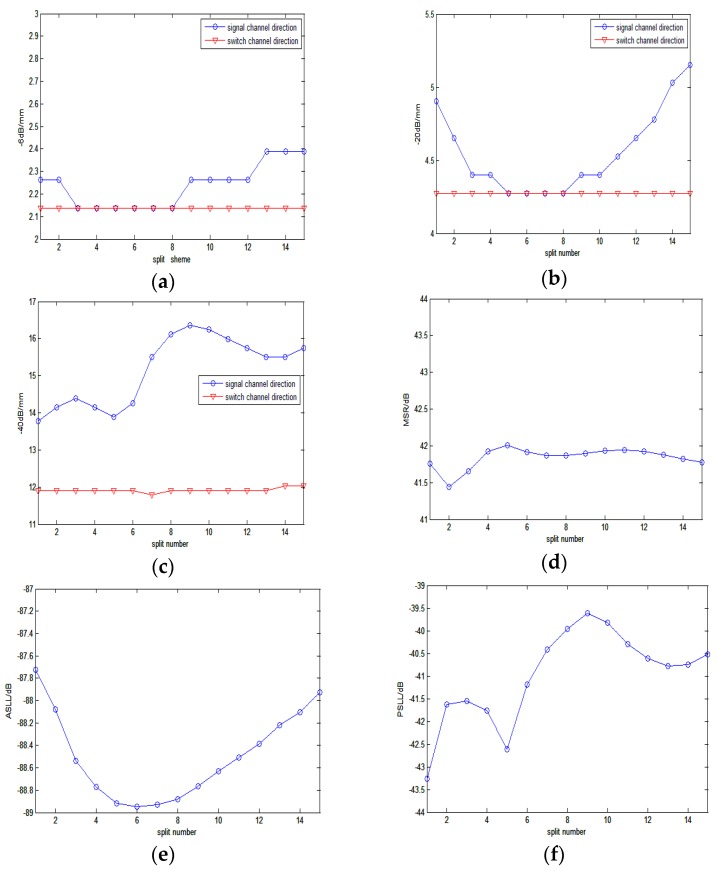
The measured parameters for different schemes. (**a**–**f**) represent the −6 dB, −20 dB, −40 dB main-lobe widths, MSR, ASLL, and PSLL vs split scheme, respectively, where the red lines and the blue lines in (**a**), (**b**,**c**) represent the −6 dB, −20 dB and −40 dB main lobe widths curves in switch channel direction and in signal channel direction, respectively.

Figure 5 shows the corresponding measured parameter curves over the split schemes. In Figure 5a–c, the −6 dB, −20 dB and −40 dB main lobe widths are presented. The blue and red curves indicate the main lobe widths in signal channel direction and switch channel direction, respectively. We can observe that the main lobe widths in the switch channel direction keep at a low value and have little change with the variation of the split scheme. This is because the synthetic-delay profile in the switch channel direction of SRCA is almost same as that of FSA. However, the main lobe widths in the signal direction have wide variations for different split schemes. For the −6 dB main lobe width in Figure 5a, scheme No. 3 to No. 8 show the best performance that the corresponding main lobe widths are the same as the switch channel direction. In addition, for the −20 dB main lobe width (Figure 5b), only schemes No. 5 to No. 8 remain to be the same as the switch channel direction. However, for −40 dB main lobe width (Figure 5c), no scheme coincides with the switch channel direction, while scheme No. 1 and No. 5 are closest. The above results indicate that scheme No. 5 can offer the best resolution. Further investigation of MSR (Figure 5d) illustrates that the maximum MSR value is also provided by scheme No. 5. Although the best ASLL (Figure 5e) and PSLL (Figure 5f) values are from scheme No. 6 and No. 1 respectively, these values of scheme No. 5 are still lower than most of other schemes (3rd of ASLL and 2nd of PSLL). This means scheme No. 5 can provide a relative superior contrast resolution.

**Figure 6:**
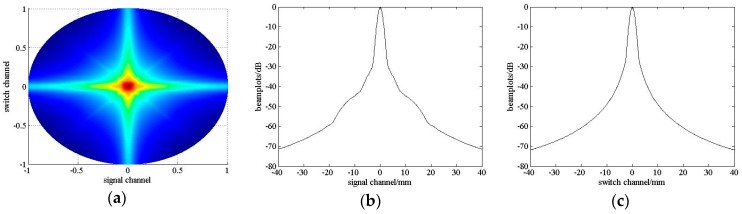

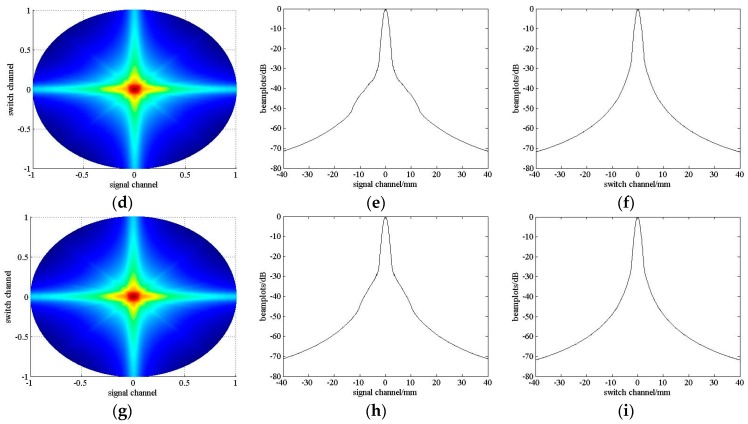
The beamplots of a 32 × 32 SRCA with K=3 for scheme No. 5**a**–**c**, scheme No. 7**d**–**f** and scheme No. 9**g**–**i** in switch channel direction and signal channel direction.

The beamplots of scheme No. 5 are showed in Figure 6 and compared with scheme No. 7 and No. 9. Table 2 lists the corresponding measured parameters. As we discussed above, the beam profiles in switch channel direction are almost the same as FSA. However, the beam profile in signal channel direction varies. Scheme No. 5 has the lowest main lobe width and highest MSR than the other two schemes. The ASLL and PSLL of scheme No. 5 are also smaller.

Taking into full consideration of the analyses above, scheme No. 5 is suggested to be the optimized one for 32 × 32 array with *K* = 3, which can provide the image quality closest to the FSA, with only 128 physical connections, while 1024 connections for FSA.

**Figure 7:**
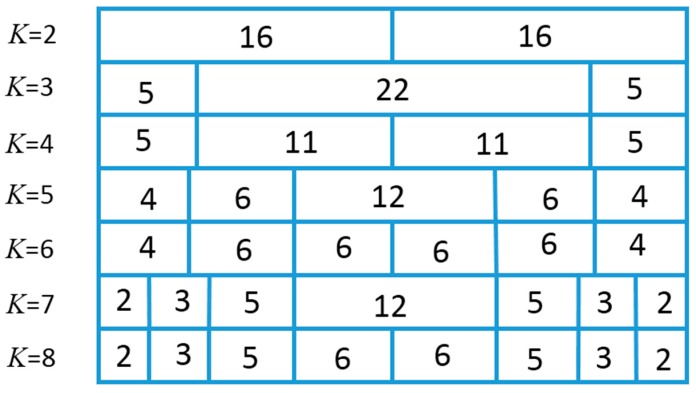
The best split scheme of SRCA with *K* from 2–8 for 32 × 32 array.

**Figure 8:**
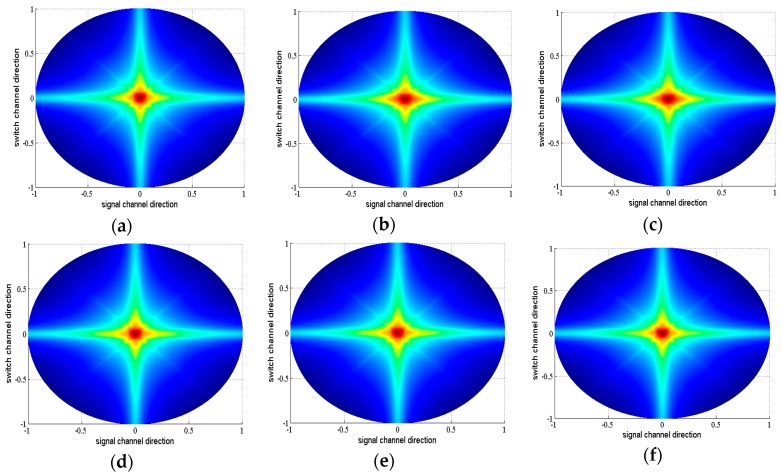

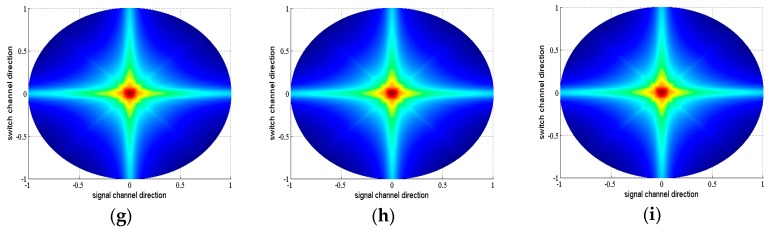
The beamplots of FSA (**a**); RCA (**b**) and best split schemes of SRCA with *K* from 2−8 (**c**–**i**) for 32 × 32 array.

Secondly, the influence of *K* to the image quality was investigated. Here, *K* valued from 2 to 8 was examined. All the possible split schemes for each *K* were tested. Due to the limit of the article length, we only list the best split scheme of each *K* (Figure 7), the corresponding beamplots (Figure 8) and measured parameters (Table 3). From Table 3, we can see that the parameters gradually tend to the FSA with the increase of *K*. When *K* = 8, the –6 dB, –20 dB and –40 dB main lobe width of SRCA are all same as those of the FSA, and MSR, ASLL, PSLL are close to those of FSA. However, a bigger *K* results in more physical connections. *K* plays a role of balancing the image quality and front-end complexity. Through analyzing the parameters in Table 3, we can find that *K* = 5 may be a suitable candidate for general applications. The –6 dB and –20 dB main lobe width is same as FSA. The –40 dB main-lobe width is only 0.53 mm bigger than that of FSA. The MSR, ASLL and PSLL differ with FSA less than 0.5 dB. This means the SRCA with *K* = 5 and the split scheme of 4_6_12_6_4 can provide image quality similar to the FSA with only 192 connections, while FSA needs 1024 connections.

**Figure 9:**
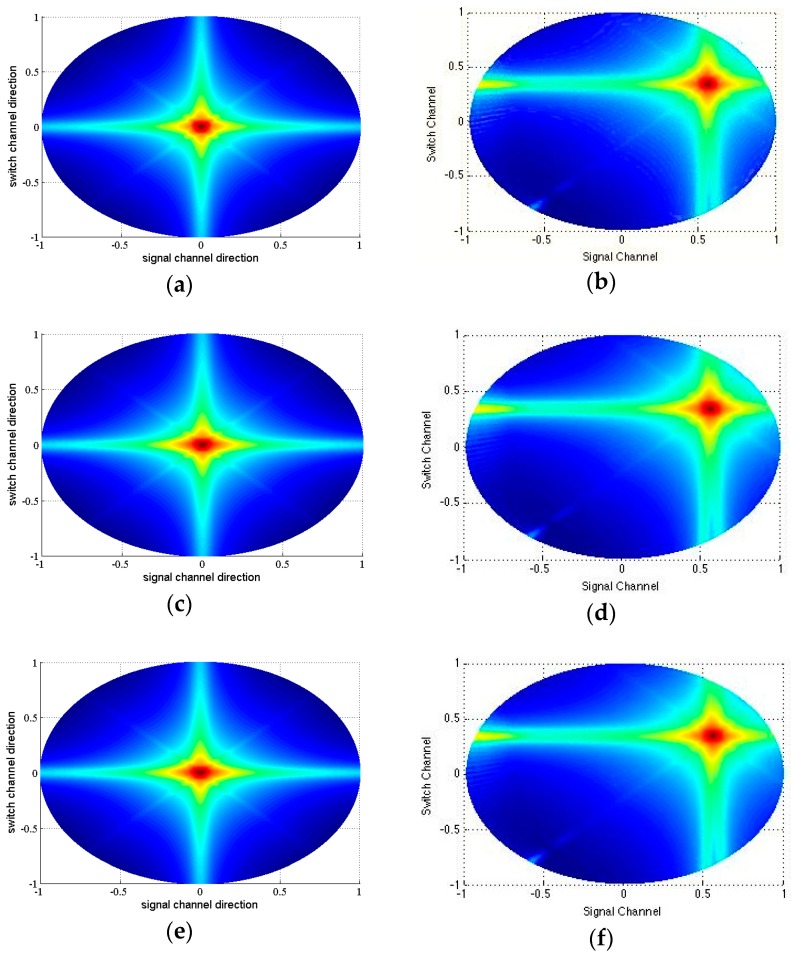
The beamplots of FSA with $\theta_{0}=0,\varphi_{0}=0$. (**a**) FSA with $\theta_{0}=45,\varphi_{0}=45$; (**b**) RCA $\theta_{0}=0,\varphi_{0}=0$; (**c**) RCA $\theta_{0}=45,\varphi_{0}=45$; (**d**) SRCA with *K* = 2 $\theta_{0}=0,\varphi_{0}=0$; (**e**) SRCA with *K* = 2 $\theta_{0}=45,\varphi_{0}=45\;$; (**f**) for 32 × 32 array.

In consideration of the off-axis profile, we revised the the Equations (6) and (7) from $\theta_{0}=0,\varphi_{0}=0$ to $\theta_{0}=45,\varphi_{0}=45$ to calculate the corresponding beam profile and the parameters. Due to the limit of the article length, we only list the best split scheme of *K =* 5, the corresponding beamplots (Figure 9) and measured parameters (Table 4).

From the Table 4, we can see that, when K = 5, the −6 dB main lobe width is the same as FSA and provide image quality similar to FSA with only 192 connections. The results also indicate that SRCA provided much further improvement than RCA even with beam-steering because SRCA could focus in the signal channel direction at the expense of increased signal channels.

Moreover, we performed point target simulation using Field II [34] to test the imaging performances of the above array designs. The point phantom had eight point targets evenly located at axial depths of 20 to 90 mm. The center frequency of the array was 4 MHz and the pitch 0.5λ. The transducer was excited by 2 periods of a sine wave at the central frequency. The single-point focus at (0,0,40) mm was applied for transmission and multi-point focuses from (0,0,20) mm to (0,0,90) mm at the interval of 10 mm for reception, respectively.

**Figure 10:**
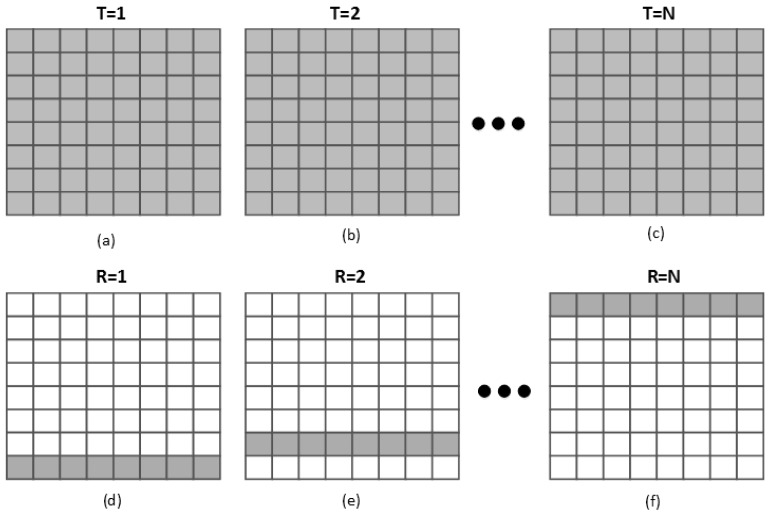
The sequence of transmit (**a**–**c**) and receive (**d**–**f**) events [19].

The sequence of transmit and receive events is illustrated in Figure 10 with an 8 × 8 array for illustrative purpose. All *N* × *N* elements are fired in transmit event simultaneously [18] (Figure 10a–c). In receive event, the echoes are collected row by row sequentially. On the first receive event, echoes are recorded by the elements in the bottom row (Figure 10d), while other rows remain inactive. On the next receive event, the next row of elements serves as the receive row (Figure 10e). This progress repeats until the top row samples the data (Figure 10f). The volume data is acquired by synthesizing all the receive data row by row.

**Figure 11:**
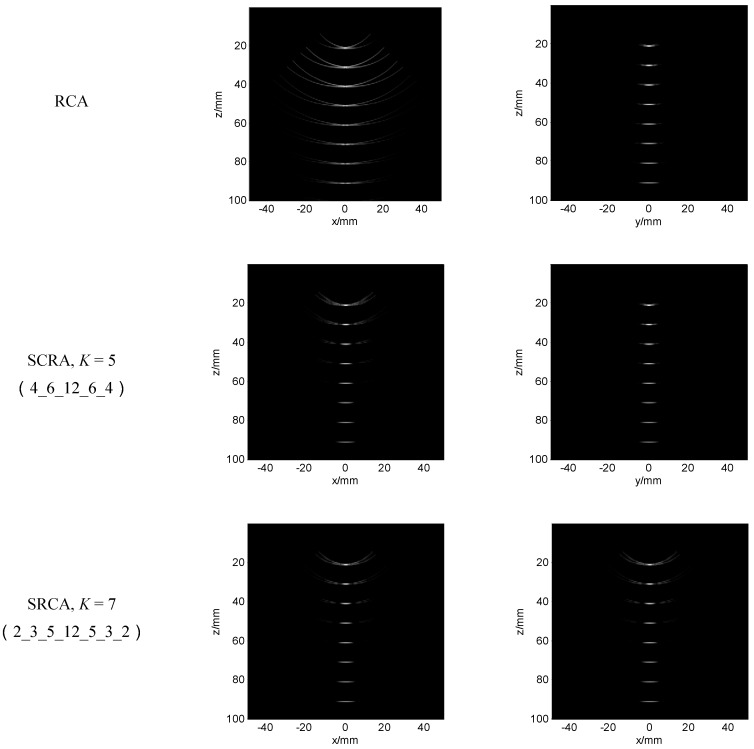
The point phantom simulated results of the different array schemes with 50 dB dynamic range. *K* is the split number and (.) is the specific split element schemes. **Left:** the signal channel section image; **Right:** the switch channel section image.

Figure 11 shows the simulation results with 50 dB dynamic range. We present two orthogonal views on x-z surface and y-z surface which correspond to the signal channel and switch channel direction, respectively. The image quality in switch channel direction for the array designs with and without splitting is similar with the FSA. In signal channel direction, RCA has large side lobes, which are reduced by SRCA with *K* = 3 (split scheme 5_22_5), especially for the targets located lower than 40 mm. It is further reduced by SRCA with *K* = 5 (split scheme 4_6_12_6_4). The image quality of SRCA with *K* = 7 (split scheme 2_3_5_12_5_3_2) is a little better than that of SRCA with *K* = 5.

## 4. Discussion

In the last section, the performance of SRCA was illustrated with a 32 × 32 2-D array. The results indicated that SRCA provided better image quality than RCA because SRCA could focus in the signal channel direction at the expense of increased signal channels. Actually, SRCA plays a tradeoff role between the RCA and the FSA. The influence of the block number *K* and the various connection schemes were also explored.

**Figure 12:**
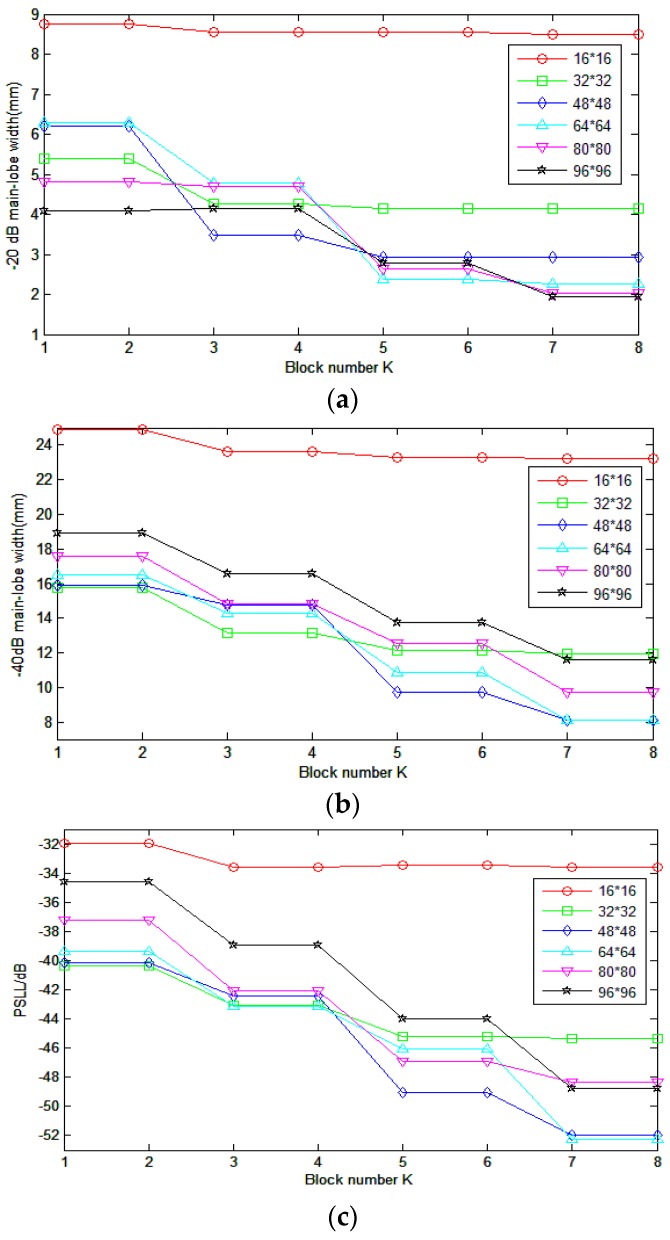
The changes of parameters for different arrays in signal channel direction. (**a**–**c**) represent the −20 dB, −40 dB main-lobe width and PSLL changed with split scheme from *K* = 1 to *K* = 8 respectively. Whereby *K* = 1 represents RCA array.

Considering that the element number of 2-D array can vary in a broad range for different applications, we also tested the performance of SRCA for different arrays: 16 × 16, 48 × 48, 64 × 64, 80 × 80 and 96 × 96. Here, only the symmetrical schemes with odd numbers of *K* were calculated as the results of adjacent even and odd numbers are similar. The best SRCA scheme of every *K* was chosen and the corresponding measured parameters in the signal channel direction are presented in Figure 12. For simplifying the experimentation, we calculated the change of –20 dB, –40 dB, and PSLL with *K* in signal channel direction. 

For 16 × 16 array, the image quality improvement by SRCA is limited. Even the difference between RCA and FSA is small, for example, the −20 dB main-lobe of the former is 8.75 mm and the latter 8.55 mm. This is mainly caused by the limited array size. For small sized array, RCA may be a good choice for the tradeoff between image quality and front-end complexity.

Similar as the 32 × 32 array, when *K* = 5, the 48 × 48 array gets close parameter results with FSA. The −20 dB, −40 dB main-lobe widths are 2.94 mm and 9.70 mm and PSLL is −49.07 dB comparing with 2.86, 7.86 mm and −52.07 dB for FSA. The corresponding splitting scheme is 5, 7, 24, 7, and 5. The connection number is only 288, one eighth of the FSA. 

As the size of the array increases, e.g., 64 × 64 array, if we still choose *K* = 5, the −40 dB main-lobe width turns to 10.85 mm which is much worse than 5.91 mm of FSA. A bigger *K* is needed for the image quality improvement. When *K* = 7 the main lobe width improves to 8.13 mm comparing to 5.91 mm of FSA. Although it still has some difficult to realize the SRCA with *K* = 7 (512 connections), it is much easier than connecting 4096 channels for FSA. 

The image qualities of 80 × 80 and 96 × 96 array splitting to 7 blocks are much lower than FSA. Searching for suitable *K* and corresponding splitting schemes for large array requires a lot of computational work and is remained for future work.

It is worth mentioning that the main-lobe width in signal channel direction for RCA can be decreased by increasing the array size. However, the side lobe level can hardly be improved. For a size specified 2-D array, SRCA can provide high image quality with relative low fabrication complexity.

In addition, SRCA has higher flexibility than RCA in transmit-receive mode design. RCA receives the echo signals by synthesizing the receive aperture row by row. Only one row can work in one receive event to avoid signals mixed. SRCA can implement various transmit-receive modes by the combination of independent channels and switches. Moreover, various kinds of apodization methods can be applied in signal channel direction in SRCA to further enhance the image quality.

## 5. Conclusions

We proposed split row-column addressing to enhance the 3-D ultrasound image quality of row-column addressing for 2-D array. SRCA achieves the compromise between the front-end complexity and the image quality. For an *N × N* array with *K* blocks, only (*K* + 1) × *N* number of interconnects are needed instead of *N × N* of FSA. For a 32 × 32 array splitting to 5 blocks, the image quality is similar to fully addressing array, but only 192 channels are needed. The fabrication complexity is reduced by about 5 times. In addition, the arrays with different sizes have been discussed. With the increase of the array size, the required blocks also increase to obtain the image quality close to FSA.

In our future work, the performance of SRCA will be verified by the hardware platform. Currently we are working on implementation of 32 × 32 array with RCA for real-time 3-D ultrasound imaging. An open platform ultrasound system has been built which can be used to realize any of the array designs. SRCA will be verified based on this open system, and more transmit-receive mode will be testified for SRCA.

## Figures and Tables

**Table 1:** The symmetrical split schemes with K=3 for a 32 × 32 array.

Split Scheme	Z_1_	Z_2_	Z_3_
1	1	30	1
2	2	28	2
3	3	26	3
4	4	24	4
5	5	22	5
6	6	20	6
7	7	18	7
8	8	16	8
9	9	14	9
10	10	12	10
11	11	10	11
12	12	8	12
13	13	6	13
14	14	4	14
15	15	2	15

**Table 2:** The measured parameters of a 32 × 32 FSA and scheme No. 5, 7 and 9 for a 32 × 32 SRCA with K=3.

32 × 32 Array	Signal Channel			Switch Channel			MSR/dB	ASLL/dB	PSLL/dB
	6 dB/mm	20 dB/mm	−40 dB/mm	6 dB/mm	20 dB/mm	−40 dB/mm			
FSA	2.1382	4.1492	11.7812	2.1382	4.1492	11.7812	43.2389	−89.4689	−45.555
SRCA No. 5	2.1382	4.2748	13.8919	2.1382	4.2748	11.9056	42.0046	−88.9165	−42.6212
SRCA No. 7	2.1382	4.2748	15.4993	2.1382	4.2748	11.9056	41.8641	−88.9310	−40.4109
SRCA No. 9	2.2639	4.4004	16.3621	2.1382	4.2748	11.9056	41.9001	−88.7653	−39.6194

**Table 3:** The measured parameters of FSA, RCA and best split scheme of SRCA with *K* from 2 to 8 for a 32 × 32 array.

32 × 32 Array	Signal Channel			Switch Channel			MSR/dB	ASLL/dB	PSLL/dB
	6 dB/mm	20 dB/mm	−40 dB/mm	6 dB/mm	20 dB/mm	−40 dB/mm			
FSA	2.1382	4.1492	11.7812	2.1382	4.1492	11.7812	43.2389	−89.4689	−45.555
RCA	2.3896	5.4048	15.7460	2.1382	4.2748	12.0300	41.7244	−87.8196	−40.3320
SRCA *K* = 2	2.3896	5.4048	15.7460	2.1382	4.2748	12.0300	41.7274	−87.8137	−40.3680
SRCA *K* = 3	2.1382	4.2748	13.8919	2.1382	4.2748	11.9056	42.0046	−88.9165	−42.6212
SRCA *K* = 4	2.1382	4.2748	13.1480	2.1382	4.2748	11.9056	42.2744	−88.9992	−43.0785
SRCA *K* = 5	2.1382	4.1492	12.1543	2.1382	4.1492	11.7812	42.7626	−89.1944	−45.1773
SRCA *K* = 6	2.1382	4.2748	12.0300	2.1382	4.1492	11.7812	42.7900	−89.2428	−45.0941
SRCA *K* = 7	2.1382	4.1492	11.9056	2.1382	4.1492	11.7812	43.1147	−89.2874	−45.3423
SRCA *K* = 8	2.1382	4.1492	11.7812	2.1382	4.1492	11.7812	43.1320	−89.3303	−45.3421

**Table 4:** The measured parameters of FSA, RCA and SRCA $\theta_{0}=45{}^{\circ},\varphi_{0}=45{}^{\circ}$ for a 32 × 32 array.

32 × 32 Array	Signal Channel	Switch Channel	MSR/dB	ASLL/dB	PSLL/dB
	−6 dB/mm	−6 dB/mm			
FSA_45_45	2.1679	2.1679	43.6788	−89.1404	−45.377
RCA_45_45	2.3913	2.1679	41.9898	−87.6176	−40.1320
SRCA_45_45 *K* = 5	2.1679	2.1679	42.7874	−89.6136	−45.1280
